# Fit for purpose: do we have the right tools to sustain NTD elimination?

**DOI:** 10.1186/1753-6561-9-S10-S5

**Published:** 2015-12-18

**Authors:** Lisa J Reimer, Emily R Adams, Mark JI Paine, Hilary Ranson, Marlize Coleman, Edward K Thomsen, Eleanor E MacPherson, T Deirdre Hollingsworth, Louise A Kelly-Hope, Moses J Bockarie, Louise Ford, Robert A Harrison, J Russell Stothard, Mark J Taylor, Nicholas Hamon, Stephen J Torr

**Affiliations:** 1 Liverpool School of Tropical Medicine, Liverpool, UK; 2 School of Life Sciences, Warwick University, Warwick, UK; 3 IVCC, Liverpool, UK

## Abstract

Priorities for NTD control programmes will shift over the next 10-20 years as the elimination phase reaches the ‘end game’ for some NTDs, and the recognition that the control of other NTDs is much more problematic. The current goal of scaling up programmes based on preventive chemotherapy (PCT) will alter to sustaining NTD prevention, through sensitive surveillance and rapid response to resurgence. A **new suite of tools and approaches** will be required for both PCT and Intensive Disease Management (IDM) diseases in this timeframe to enable disease endemic countries to:

**1. Sensitively and sustainably survey** NTD transmission and prevalence in order to identify and respond quickly to resurgence.

2. Set relevant control targets based not only on epidemiological indicators but also entomological and ecological metrics and use **decision support** technology to help meet those targets.

3. Implement **verified and cost-effective tools** to prevent transmission throughout the elimination phase.

Liverpool School of Tropical Medicine (LSTM) and partners propose to evaluate and implement existing tools from other disease systems as well as new tools in the pipeline in order to support endemic country ownership in NTD decision-making during the elimination phase and beyond.

## Background

With increased financial support and international political momentum, the goals for neglected tropical diseases (NTDs) control and elimination are changing. Over the next five years we can expect disease-appropriate scale up of community mass drug administration, intensified case detection and management and scaling up vector control [1]. Research is underway to integrate NTDs into the health system, deliver care to neglected communities and to optimize testing and treatment regimes. Improvements in delivery and increased access will enable country programmes to meet their targets [2] and over the next ten years we expect many countries to have successfully reached the WHO Roadmap objectives and the London 2020 goals for the eliminable and eradicable NTDs [1,3] and enter the “endgame”, i.e. the final stages of elimination campaigns where disease is still present at reduced levels [4]. During the endgame programme priorities will shift from increasing intervention coverage in order to meet elimination targets, to scaling down certain interventions and sustaining elimination for the long term through surveillance and targeted control.

## Unique challenges facing control programmes during the endgame

The elimination goals and timelines, as well as the control approaches to reach these goals are as diverse as the 17 NTDs themselves. Lessons from malaria elimination efforts have revealed heterogeneities in the stability of sustained elimination due to a multitude of factors including differences in transmission potential, reproductive rate, cross border activity, socioeconomic status, baseline prevalence and human behaviour [5]. However, while it is impossible to draft a single elimination approach for one disease, much less all NTDs, we can begin preparing for the new and unique challenges likely to face NTD programmes in the endgame. The employment of existing, community-wide strategies when prevalence is low will no longer be cost-effective or epidemiologically justified and instead we must prepare for the challenge of sustaining surveillance and control following the anticipated post-2020 scale down. Failure to reach elimination thresholds during the endgame will compromise neighbouring and regional NTD elimination; however what constitutes elimination is poorly defined and elimination targets remain untested.

## Emerging priorities for control programmes

As country programmes move towards the 2020 milestones and targets, they will require a new suite of tools and approaches in order to sensitively assess transmission intensity and provide an evidence base for the appropriate stage to cease community wide treatments [6]. The tools and approaches that will lead to success in meeting 2020 targets are not necessarily suitable for the endgame. Successful and sustainable elimination will hinge on a country programme's ability to 1) identify transmission and transmission risks; 2) decide how, when and where to respond and 3) deliver optimum interventions.

## What LSTM can deliver

**Figure 1:**
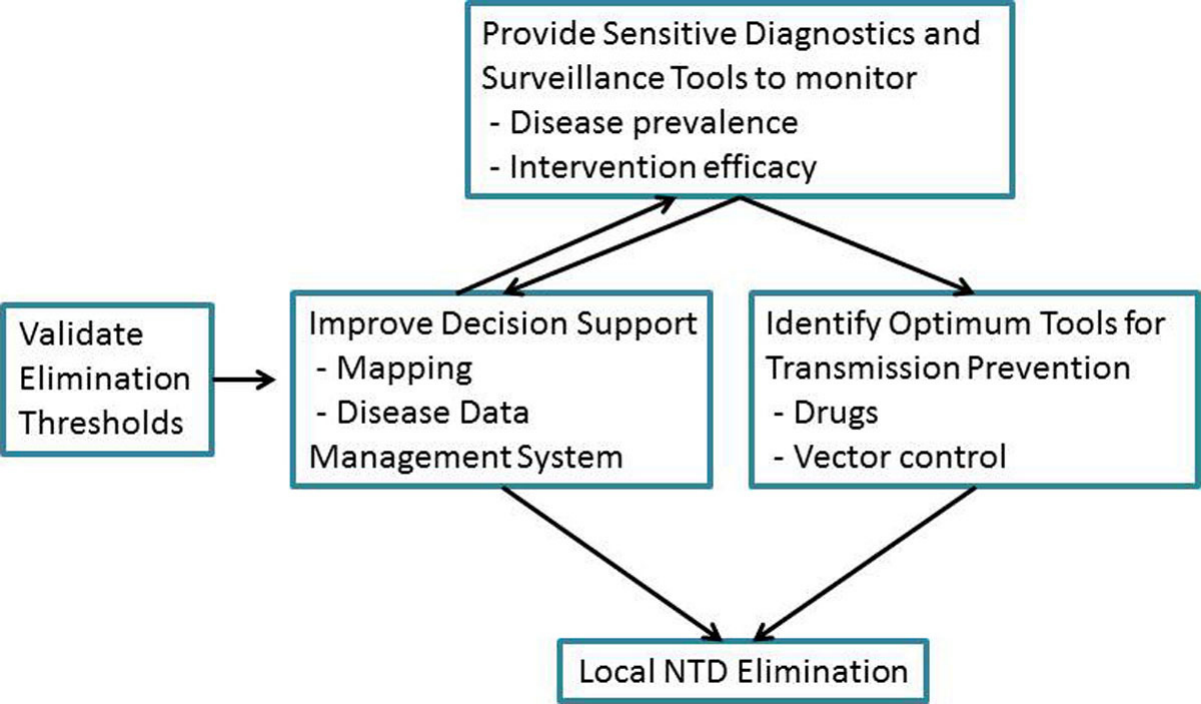
Proposed research outputs to directly support NTD programmes in sustaining elimination

LSTM, in collaboration with many international partners, has played a pivotal role in research to inform NTD control; from the development and evaluation of new tools for elimination, mapping and surveillance to implementation research, capacity building and technical assistance. We are well poised to deliver research on how best to support country programmes through the next phase by readying the tools, technologies and approaches needed in response to the above emerging needs. Specifically we propose to support elimination programmes in surveillance, decision making and transmission prevention (Fig 1).

## Surveillance

### Improved diagnostics and approaches to disease surveillance

As we strive and reach for elimination in NTDs the diagnostic requirements will change and there will be an increased need for disease surveillance, identification of foci and collection of data to inform decision making. LSTM's Research Centre for Drugs and Diagnostics (RCDD) is working with industry (SMEs and larger organisations), academia and other NGOs to discover, develop and deliver novel drugs and diagnostics against major human pathogens to meet these needs.

We anticipate that the PCT diseases will require large-scale mapping and screening of patients in order to identify hotspots of disease. It is essential that surveillance is integrated into existing health system structures in order that sustained monitoring can take place. LSTM is working on molecular diagnostic surveillance systems that will work hand-in-hand with existing networks such as the Global Polio Eradication Initiative and Malaria Elimination Initiative.

The IDM NTDs such as HAT, leishmaniasis and dengue will require sensitive diagnostics that are able to pick up asymptomatic and early infection, identify drug resistance, monitor patient response and inform patient care. Snakebite is an additional IDM NTD with specific challenges that include improved diagnostics as a tool for both clinical management and assessing disease burden. To enable decentralisation, testing devices should be portable and require only moderately invasive sampling methodologies. LSTM are developing and/or evaluating diagnostics such as LAMP, GeneXpert, Genedrive for use in elimination settings. In the medium to long-term RCDD will push for innovative diagnostic solutions that will be close to the patient to inform treatment, patient care and the ability to store data and transmit immediately to inform disease management tools.

### Tools to monitor intervention efficacy and resistance

With insecticides playing an increasingly important role in the elimination of vector-borne NTDs [1,6], it has become necessary to monitor the emergence and spread of insecticide resistance. LSTM has led the field in evaluating intervention efficacy of insecticides for vector-borne diseases and insecticide resistance detection and management [7-9]. A range of new tools are becoming available over the next two years which we will evaluate for NTD vector control operations. Such tools include new field-friendly assays to track the actual amounts of insecticides being sprayed or used in nets, as well as new methods to track the genes involved in resistance [10]. Integrating these into appropriate NTD vector control operations could have game changing potential in preventing transmission through effective use of insecticides and tight monitoring of resistance.

For instance, the Insecticide Quantification Kits (IQK), which are simple low cost assays that can be used on-site to measure insecticides used for house spraying and bednets, are now commercially available for carbamate and cyanopyrethroid monitoring. Kits for DDT and organophosphate monitoring are expected to be available in 2015. These kits will allow for internal validation of insecticide-based control and enable programmes to optimize the impacts of available tools. The IQK is currently being adapted for the VL elimination programme in India, and we propose further adapting it for monitoring Chagas and dengue programmes. Vector population monitoring tools have been developed for malaria mosquitoes to identify mosquito species, malaria infection status and resistance mutations. This information is a key part of the data needed by programme managers to evaluate transmission impacts and mitigate potential resistance. The genomic data accruing on NTD vectors can be readily exploited to develop DNA based vector monitoring tools that parallel those now being routinely applied for monitoring malaria vectors.

Finally, a new generation of generic ‘predictive probes’ that are capable of identifying genes with insecticide resistance potential have been recently developed [11]. These allow the development of ultra-early warning systems to detect incipient resistance at a point that something sensible can be done to prevent its spread. We intend to translate these into practical insecticide resistance monitoring tools across the spectrum of NTD vectors. The availability of tools to monitor the impact of interventions within the programme will allow for a rapid adjustment in either the elimination tool or approach in order to reach programme targets.

## Decision support

### Mapping to prioritize surveillance and coverage

Mapping prevalence data and the impact of single or multiple interventions over time and space is critically important to improve decision making for NTD programmes. We have developed novel mapping methods to address the co-endemicity and overlapping interventions to help determine the risks and benefits of different intervention and surveillance strategies. The first we designate as micro-stratification overlap mapping (MOM) which focuses on the overlapping distribution of infections or co-endemicity in a particular geographical area at a finer spatial resolution. The MOM approach is essential for planning the expanded distribution of drugs in countries co-endemic for filarial infections, as has been demonstrated in the Democratic Republic of Congo (DRC) and Nigeria [12,13]. The second mapping method focuses on quantifying the overlap and potential impact of multiple interventions at subnational level to produce a multiple intervention score map (MISM) [14]. The MISM approach aims to develop practical programmatic maps to assist NTD programme managers to identify and target high-risk areas that may not have received adequate impacting interventions to interrupt the transmission of the disease. This is critical for countries as they prepare to scale down coverage and enter the elimination phase as exemplified by the LF Elimination Programme in Malawi [14]. In addition, geo-spatial models have been developed to produce baseline risk maps, incorporating socio-demographic and environmental factors, with the aim of overlaying intervention coverage and predicting areas where transmission is mostly likely to persist and in need of alternative or enhanced intervention strategies [15].

### Disease Data Management Systems (DDMS)

Underpinning the successful elimination of NTDs will be access to information. Masses of information on disease, interventions, and vectors are being collected by control programmes but are not mined to improve decision-making. Countries will require tools that allow 1) easy access to a central data repository and 2) interpretation of that data to guide decisions on how to achieve the greatest impact with limited resources. Informed decision-making will also allow programmes to identify priority areas for surveillance as well as when and where to implement further control measures.

LSTM, with support from the Innovative Vector Control Consortium (IVCC), has been instrumental in developing and implementing a sophisticated Disease Data Management System (DDMS) in endemic countries. At its core, the DDMS is an open-source database for controlling programme data, from case surveillance to entomological monitoring to intervention data. However, there are several features that make the system unique. First, it is integrated with mapping and reporting tools, allowing programmes to use their data to support real-time informed decision-making. Second, it has customizable thresholds that will send alerts when targets are not being met. Third, it is flexible enough to switch between several vector borne diseases. Current modules include dengue, malaria and most recently visceral leishmaniasis (VL).

Malaria modules have been implemented in Zambia, Ethiopia, and Equatorial Guinea. The Zambian National Malaria Control Centre and Zambia Integrated Systems Strengthening Program have worked closely together to use the system to monitor entomology and intervention data with technical support from LSTM. Work is underway to integrate the DDMS with VL elimination efforts in Bihar, India, enabling the country to track insecticide resistance patterns, IRS coverage and quality, and case surveillance data. Over the next 10-20 years, programmes will transition from control to elimination, involving a scale down from community-level to targeted interventions. The DDMS is poised to facilitate this transition and will be further developed to ensure that programmes have access to critical information that support decision-making.

### Entomological and epidemiological research to validate transmission endpoints for NTD elimination

Elimination targets remain untested and many programmes assume that a certain number of treatments or a given number of MDA rounds will be sufficient to interrupt transmission [6] of PCT diseases. Current assumptions of threshold biting rates, and threshold parasite breakpoints, below which transmission cannot be sustained, are largely untested. It is essential that these assumptions are validated to ensure elimination targets are locally relevant.

For example, our research on vector-borne NTD transmission has shown that differences in vector competence are likely to influence elimination success [16]. Further work, in collaboration with the NTD modelling group, will refine transmission thresholds. Research to validate elimination targets will involve experimental vector work to determine transmission potential, modelling to predict threshold inoculation rates sufficient for resurgence [17], followed by observational studies throughout the elimination phase. With sufficient evidence, the DDMS can be programmed to include updated, relevant and realistic elimination targets. This will allow programmes to prioritize resources and gain confidence that intervention efforts will be successful.

## Tools to prevent transmission

### Evaluate existing tools and their application to NTDs

Vector control is a fundamental element of the global strategy to fight malaria, with a proven track record of successfully reducing disease transmission. Indoor residual spraying (IRS) and long-lasting insecticidal nets (LLINs) are the two core malaria vector control measures broadly applicable to indoor biting vectors. Given their success in malaria operations they are strongly recommended for integration in NTD programmes with appropriate vectors (e.g. LF, VL, Chagas) [18]. Bednets have a proven role in reducing snakebite incidence [19] and this additional health benefit could be readily implemented into integrated NTD management. Other vector-borne NTDs (HAT, dengue, onchocerciasis, LF in Pacific) are more difficult to integrate with existing programmes because the vectors require different approaches that are not currently used at scale. Existing vector-based interventions are proven to impact the vector population and some recent studies have demonstrated direct impacts on disease prevalence [20]. However, evidence is lacking to date on the direct impact of vector control on the prevalence of many NTDs and the longer term contribution to elimination.

Before recommending that available interventions be tailored to and adopted by NTD programmes, we propose to critically evaluate their impact on NTD transmission and prevalence. For many of the NTDs (e.g. with longer periods of latency), this will require extended periods of surveillance to ensure that vector population reductions closely correspond with decreases in incidence over time. We will build on our current expertise in vector control for NTDs [21-25] and work with our partners to strengthen the evidence base of the available tools for NTD control.

### Ability to evaluate suitability of new tools in the pipeline for use in NTDs

A thorough evaluation of available and emerging chemotherapies, diagnostics and vector control tools, mapping and decision making systems will allow programmes to decide which interventions are likely to give the desired impact, while also providing additional tools at the ready if efficacy is compromised.

New chemotherapies are emerging for onchocerciasis, LF and HAT [26-28] which will play an integral role in case detection and treatment through the endgame. The Anti-*Wolbachia* Consortium (A·WOL; http://www.a-wol.com), a drug discovery and development programme [29], was founded at LSTM in 2007 to screen, identify and validate novel drugs as well as develop treatment regimens with existing macrofilaricidal drugs, e.g. doxycycline, which has been adopted for use by elimination programmes as an alternative strategy in areas of *Loa loa* co-endemicity and at risk of severe adverse events (SAE) to ivermectin [26]. The benefits of an anti-*Wolbachia* macrofilaricidal drug would be to 1) reduce programme time frames, 2) provide an alternative treatment to existing drugs with reduced efficacy, 3) be useable in *Loa loa* co-endemic areas without risk of SAE, 4) improve morbidity management, and 5) complement existing strategies for MDA end-game.

In addition to the A·WOL approach to macrofilaricidal drug development, other strategies aim to discover and develop macrofilaricidal drugs which act by directly killing adult worms [27] with other candidates emerging from the drug discovery pipeline. We propose to further evaluate the suitability of emerging drugs for use in the endgame and in areas where existing strategies are compromised.

Diagnostic evaluation in well-designed trials is essential prior to and during implementation as prevalence and endemic settings change. LSTM has several multi-disease diagnostic evaluation sites including Nigeria, Ethiopia and Malawi where the suitability of tests can be appraised. Our Clinical Sciences unit is highly skilled and qualified in the implementation of diagnostics and their continued evaluation, suitability, cost-effectiveness and roll-out.

The IVCC, established at LSTM in 2005, seeks to overcome the barriers to innovation in the development of vector control tools. It is supporting the development of novel insecticides, long-lasting formulations, and delivery mechanisms that will improve the efficacy of chemical control. Currently, there are eight novel classes of chemistry under evaluation, and three new active ingredients will be brought to market to act as alternatives to pyrethroids and other chemical classes that are now showing resistance. Improvements in the residuality of indoor residual sprays via new products such as Bayer's new polymer-enhanced formulation of deltamethrin and Syngenta's primiphos-methyl CS formulation have been rolled out, and combination nets that incorporate synergists or multiple insecticides are being evaluated to overcome pyrethroid resistance [10].

## Conclusion

The shift in priorities and the emergence of new challenges as national NTD programmes approach the endgame will require a new suite of tools and approaches to sustain elimination. Many tools for surveillance, decision making and long term transmission prevention are in the pipeline or are currently being successfully applied in other disease control programmes. We must prioritise the evaluation, application and implementation of these tools in order to support NTD programmes in reaching and sustaining their elimination targets.

## Competing interests

The authors declare that they have no competing interests

## Authors' contributions

All authors contributed to the development of the final manuscript.

## Declarations

This article has been published as part of *BMC Proceedings* Volume 9 Supplement 10, 2015: The 2014 CAHRD (Collaboration for Applied Health Research & Delivery) Consultation. The full contents of the supplement are available online at http://www.biomedcentral.com/bmcproc/supplements/9/S10.
